# Cataract surgery causes biomechanical alterations to the eye detectable by Corvis ST tonometry

**DOI:** 10.1371/journal.pone.0171941

**Published:** 2017-02-21

**Authors:** Yoshitake Kato, Shunsuke Nakakura, Ryo Asaoka, Kanae Matsuya, Yuki Fujio, Yoshiaki Kiuchi, on behalf of the Ocular Biomechanics Study Group

**Affiliations:** 1 Department of Ophthalmology, Saneikai Tsukazaki Hospital, Himeji, Japan; 2 Department of Ophthalmology, The University of Tokyo, Tokyo, Japan; 3 Department of Ophthalmology and Visual Science, Hiroshima University, Hiroshima, Japan; Medizinische Universitat Graz, AUSTRIA

## Abstract

**Purpose:**

Modern cataract surgery is generally considered to bring about modest and sustained intraocular pressure (IOP) reduction. However, the pathophysiological mechanism for this remains unclear. Moreover, a change in ocular biomechanical properties after surgery can affect the measurement of IOP. The aim of the study is to investigate ocular biomechanics, before and following cataract surgery, using Corvis ST tonometry (CST).

**Patients and methods:**

Fifty-nine eyes of 59 patients with cataract were analyzed. IOP with Goldmann applanation tonometry (IOP-G), axial length, corneal curvature and CST parameters were measured before cataract surgery and, up to 3 months, following surgery. Since CST parameters are closely related to IOP-G, linear modeling was carried out to investigate whether there was a change in CST measurements following cataract surgery, adjusted for a change in IOP-G.

**Results:**

IOP-G significantly decreased after surgery (mean±standard deviation: 11.8±3.1 mmHg) compared to pre-surgery (15.2±4.3 mmHg, P<0.001). Peak distance (the distance between the two surrounding peaks of the cornea at the highest concavity), maximum deformation amplitude (the movement of the corneal apex from the start of deformation to the highest concavity) and A1/A2 velocity (the corneal velocity during inward or outward movement) significantly increased after cataract surgery (P<0.05) while radius (the central curvature radius at the highest concavity) was significantly smaller following cataract surgery (P<0.05). Linear modeling supported many of these findings, suggesting that peak distance, maximum deformation amplitude and A2 velocity were increased, whereas A2 deformation amplitude and highest concavity time were decreased (after adjustment for IOP change), following cataract surgery.

**Conclusion:**

Corneal biomechanical properties, as measured with CST, were observed to change significantly following cataract surgery.

**Trial registration:**

Japan Clinical Trials Registry UMIN000014370

## Introduction

Cataract is one of the most common ophthalmic diseases among elderly people and was reported to be the leading cause (33.4%) of global blindness in 2010 [1]. Cataract surgery results in the recovery of visual acuity, but also induces a short-term effect of lowering intraocular pressure (IOP) in normal subjects [2] as well as glaucoma patients [3].

However, the effects of cataract surgery on the ocular environment, such as the aqueous humor or vitreous cavity remain unknown. Additionally the mechanism of IOP reduction after cataract surgery remains poorly understood and the magnitude of this effect is highly variable and unpredictable. Hsu et al. reported that the percentage of IOP reduction after cataract surgery–in non-glaucomatous eyes with open angles–is greater in eyes with anteriorly-positioned lenses [4]. The difference in lens position may suggest a difference in the biomechanical properties of the eye, which may, in fact, drive IOP changes after cataract surgery. Additionally, elevated levels of pro-inflammatory

cytokines like monocyte chemoattractant protein-1 in aqueous humor associate with IOP reduction after cataract surgery [5]. Indeed, in some cases, a measured decrease in IOP after cataract surgery may not reflect a true change in IOP, but are due to a change in the biomechanical properties of the eye that include corneal properties but also supporting structures, like sclera, that compose the mechanical boundary of the cornea [6]. The effect of cataract surgery on ocular biomechanical parameters has simply not been investigated in great detail. Previous reports, however, have demonstrated changes in corneal hysteresis (CH) and corneal resistance factor (CRF), measured by the Ocular Response analyzer, after cataract surgery [7, 8]. Kamiya et al. reported that both CH and CRF decreased one day postoperatively, however, these parameters recovered to their baseline values at 1 week and 3 months after surgery [7]. On the other hand, Kucumen et al. reported that CH and CRF were decreased 1 week following surgery, but recovered to their baseline values 1 month and 3 months after surgery [8]. Nevertheless, CH and CRF cannot fully explain biomechanical changes to the cornea [9].

Recently, it has been shown that a non-contact tonometer with a high speed camera can detect highly-detailed corneal movements, following applanation by a rapid air puff [10–12]. The Corneal Visualization Scheimpflug Technology tonometer (Corvis ST tonometry: CST, Oculus, Wetzlar, Germany) is equipped with a high-speed camera (4330 frames per second) that can capture a series of horizontal Scheimpflug images during corneal deformation with an air puff jet [13–15]. The instrument also provides many detailed biomechanical parameters. These measurements are capable of differentiating corneal biomechanics between glaucoma subjects and normal subjects [16–18], the relation between glucose control in diabetes mellitus and their corneal biomechanics [19], characteristics of corneal diseases [20, 21] and changes in ocular biomechanics after corneal refractive surgery [22, 23]. CST provides twelve corneal biomechanical parameters that describe different properties of corneal deformation after the air puff applanation. One previous report investigated corneal biomechanics before and after cataract surgery using CST [24], however, the precise mechanism for the observed change in biomechanics remains unclear. The first aim of the present study was to confirm a change in ocular biomechanics following cataract surgery, using CST. The second aim of our study is to explore the mechanism for these biomechanical changes to the eye using linear modeling, with an appropriate adjustment for IOP change.

## Patients and methods

This was a prospective, observational study that was approved by the Research Ethics Committee of Saneikai Tsukazaki Hospital (Himeji, Japan), the Graduate School of Medicine and Faculty of Medicine at The University of Tokyo (Tokyo, Japan), and Hiroshima University Hospital (Hiroshima, Japan).

Written consent was provided by all patients for their information to be stored in the hospital database and used for research. This study was performed according to the tenets of the Declaration of Helsinki.

### Patients

Inclusion criteria were patients with cataract and those scheduled for cataract surgery. Exclusion criteria were subjects with glaucoma, ocular hypertension, retinal disease, corneal disease, any prior intraocular surgery, or those who experienced any specific complications after cataract surgery, including secondary high intraocular pressure (IOP) elevation or severe inflammation. Patients with suspected occludable angle were evaluated with gonioscopy by each doctor.

Sixty four cataract eyes of 64 patients were prospectively enrolled at Saneikai Tsukazaki Hospital and Hiroshima University Hospital between the period of June 2014 and November 2015. Five patients were excluded because of incomplete data (2 patients), low quality data in CST (2 patients) and secondary high IOP elevation after cataract surgery (1patient). Finally, 59 eyes of 59 patients were included in the analysis.

The mean age was 74.0±7.1 [60–90] (mean ± standard deviation: SD [range]) years old, and 32 males and 32 right eyes were included.

All patients had undergone uneventful cataract surgery (phacoemulsification cataract extraction with posterior chamber intraocular lens (IOL) implantation) by a 2.8 mm temporal corneal incision (37 eyes of 37 patients) or a 2.8 mm superior sclera-corneal incision (22 eyes of 22 patients). When both eyes of a patient met the inclusion criteria, one eye was randomly chosen.

Data were obtained prospectively at pre-surgery (pre) as well as 1 week (1w), 1 month (1m) and 3 months (3m) after cataract surgery. After surgery, steroidal (0.1% Fluorometholone Ophthalmic Solution, fluorometholone; Santen, Osaka, Japan) and antibiotic (1.5% Cravit, levofloxacin; Santen, Osaka, Japan) medications were topically administered 4 times a day for 1 month, and nonsteroidal anti-inflammatory (0.1% Nevanac, nepafenac ophthalmic solution; Alcon, Tokyo, Japan) medication was also topically administered 3 times a day for 1 month in all cases.

Goldmann applanation tonometry (GAT) remains the gold standard for measuring IOP. Therefore, in the current study, GAT was used to measure IOP (IOP-G); three separate measurements were carried out at each time point and the average value was used in the analyses. Additionally, three sets of IOPs were measured using the non-contact tonometer CT-90A (Topcon, Tokyo, Japan).

Axial length (AL) was measured five times using the IOLMaster, ver. 5.02 (Carl Zeiss Meditec, Jena, Germany), and the mean of the five measurements was used in subsequent analyses. The horizontal (K1) and vertical (K2) corneal curvature were also measured using the IOLMaster, and the average of K1 and K2 (Kerato) was used in the analyses.

### Corvis ST tonometer measurements

Measurements with CST (Ver.1.02r1092) were performed three times at each time point and the average of the three measurements was used in statistical analyses. The principles of CST have been described in detail elsewhere [13–24]. In short, CST is a non-contact tonometer with a high-speed Scheimpflug camera (capturing 4,330 frames per second) to monitor the corneal response to an air puff pulse that forces the cornea inward until it reaches a concavity phase. The ‘A1/A2 deformation amplitude’ and ‘HC deformation amplitude’ are unique parameters in the newest software of CST (Ver.1.02r1092). ‘HC deformation amplitude’ in this version of software is identical to the ‘maximum deformation amplitude’ in older software versions, therefore we have used this previous and more popular term [13–24].

**Fig 1:**
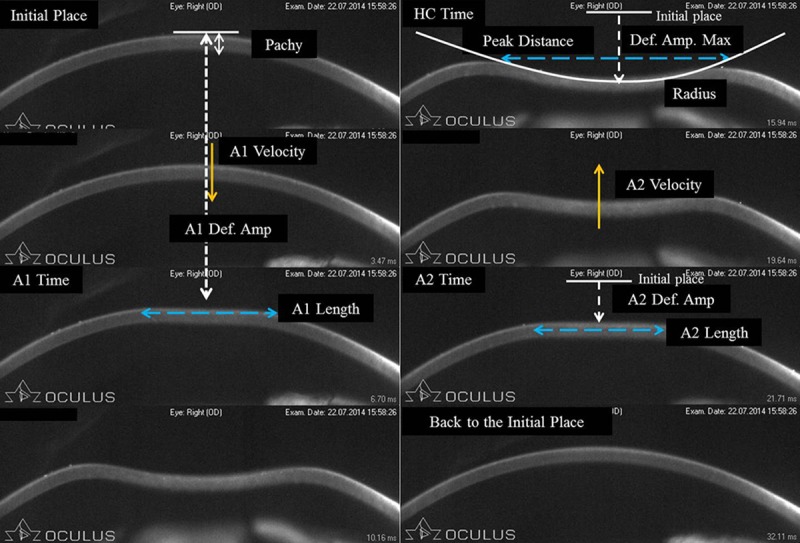
Corneal movements after an air puff from CST and obtained parameters. Pachy: central corneal thickness; A1/A2 Time: the durations of time taken from initiation of the air puff to first (when the cornea is moving inward) or second applanation (when the cornea moves outward); A1/A2 Length: the lengths of the flattened cornea at first and second applanations; A1/A2 Velocity: the corneal velocities during first and second applanations; HC Time: the length of time from the initiation of the deformation to the point when the cornea reaches highest concavity; Radius: the central curvature radius at the highest concavity; Peak Distance: the distance between the two surrounding peaks of the cornea at the highest concavity; A1 Def. Amp: the moving distance of the corneal apex from the initial position to that at the A1 Time; A2 Def. Amp: the moving distance of the corneal apex from the initial position to that at A2 Time; Def. Amp. Max: the distance of the corneal apex movement from the initiation of the deformation to the highest concavity.

Fig 1 shows the 13 parameters provided by CST with the latest software.

Pachy indicates the central corneal thickness (CCT); ‘A1/A2 Time’ are the durations of time taken from initiation of the air puff to first (when the cornea is moving inward) or second applanation (when the cornea moves outward); ‘A1/A2 Length’ are the lengths of the flattened cornea at first and second applanations; ‘A1/A2 Velocity ‘ are the corneal velocities during first and second applanations; ‘highest concavity time (HC Time)’ is the length of time from the initiation of the deformation to the point when the cornea reaches highest concavity; ‘radius’ is the central curvature radius at the highest concavity; ‘peak distance’ is the distance between the two surrounding peaks of the cornea at the highest concavity; ‘A1 Def. Amp’ is the moving distance of the corneal apex from the initial position to that at the A1 Time; ‘A2 Def. Amp’ is the moving distance of the corneal apex from the initial position to that at A2 Time; ‘maximum deformation amplitude (Def. Amp. Max)' is the distance of the corneal apex movement from the initiation of the deformation to the highest concavity (note that the movement of the corneal apex is compensated by the movement of the whole eye [14]).

All measurements were conducted between 1:00 PM and 4:00 PM in the afternoon to avoid circadian changes in IOP and ocular biomechanical properties.

### Statistics

The Tukey-Kramer test was used to detect statistically significant differences among the CST parameters as well as IOP-G and CT-90A. Since CST parameters are closely related to IOP-G [14, 25] and other clinical factors [14, 25], the effect of cataract surgery on a given CST parameter should be evaluated using a statistical model that makes an appropriate adjustment for IOP-G and any other confounding variables. Hence the effect of cataract surgery on each CST parameter was investigated based on a starting model that included 7 variables: age, sex, IOP-G, AL, CST-determined CCT, Kerato and status of eye (pre-surgery or 3m post-operation); the optimal linear model was then selected from among all possible combinations of the predictors (2^7^), according to the second-order bias-corrected Akaike Information Criteria statistic (AICc). The degrees of freedom in a multivariate regression model decreases with a large number of variables. Therefore, the use of model selection methods is recommended to improve model fit by removing redundant variables rather than simply applying a multivariable regression model using all variables [26, 27]. The AIC is a common statistical measure used in model selection, and the AICc is a corrected version of the AIC which gives an accurate estimation even when the sample size is small [26].

In addition, the magnitude of change in CST parameters between the corneal incision group (N = 37) and sclera-corneal incision group (N = 22) was evaluated by Welch’s t-test. Statistical analyses were carried out using the JMP, version 10.0.0, software package (SAS Institute Inc., Cary, NC) and the statistical programming language ‘R’ (R version 3.1.3; The Foundation for Statistical Computing, Vienna, Austria). Values of p < 0.05 were considered to be statistically significant.

## Results

**Fig 2:**
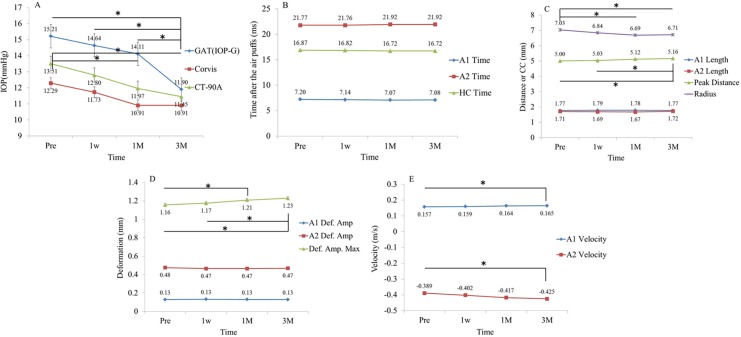
Changes in values of IOP-G, CT-90A and CST parameters following cataract surgery. *: indicates a significant difference according to the Tukey-Kramer test. Values are expressed as mean plus error.

Fig 2 illustrates the changes in IOP-G and CST parameters following cataract surgery. As shown in Fig 2A, IOP-G at 3m (11.8±3.1 mmHg) was significantly lower than pre (15.2±4.3 mmHg), 1w (14.6±3.9 mmHg), and 1m (14.1±3.7 mmHg) according to the Tukey-Kramer test (p<0.05). Similarly, IOP-CT90A readings at 1m (11.9±2.9 mmHg) and 3m (11.4±2.7 mmHg) were significantly lower than pre (13.5±3.2 mmHg) measurements (p<0.05, Tukey-Kramer test). No statistically significant differences were found in CST IOP values between pre, 1w, 1m and 3m (Tukey-Kramer test, p>0.05). As shown in Fig 2B, no significant differences were observed for A1 Time, A2 Time or HC Time. Fig 2C reveals that no significant differences were found for A1 or A2 Length, but peak distance was significantly higher at 3m compared with pre and 1w, and radius values were significantly smaller at 1m and 3m compared with pre. Fig 2D shows that no significant differences were found in A1 or A2 deformation amplitudes but maximum deformation amplitude at 3m was significantly higher than at pre and 1w, and maximum deformation amplitude at 1m was significantly higher than that at pre. Finally, Fig 2E demonstrates a significant difference between A1 and A2 velocity at pre and 3m.

Overall, after cataract surgery and the associated IOP reduction, the cornea will be deformed more quickly (A1 Velocity is increased) and more deeply (maximum deformation amplitude is increased) after the air puff. Furthermore, at the point in time that the cornea is maximally deformed cornea (HC Time), the cornea is deformed more widely (peak distance is increased) and more steeply (radius is decreased), however, after that the cornea will accelerate back to its initial shape (A2 Velocity is increased).

Axial length and CCT were not significantly different at any time point (AL: 23.60 mm at pre, 23.51 mm at 1w, 23.52 mm at 1m and 23.51 mm at 3m; CCT: 554 mm at pre, 554 mm at 1w, 549 mm at 1m and 545 mm at 3m).

### Change in CST parameters pre- vs. 3m post-cataract surgery

As shown in Table 1, status (whether a measurement was taken pre- or 3m post-operation) was selected in the optimal models to describe: A2 Velocity (coefficient = -0.023 for 3m-post operation), A2 deformation amplitude (-0.023), HC Time (-0.151), maximum deformation amplitude (0.032), and peak distance (0.080). A2 Velocity, maximum deformation amplitude and peak distance increased after cataract surgery whereas was A2 deformation amplitude and HC Time decreased following surgery; these changes appear to be independent from IOP change.

**Table 1:** Parameters selected in the optimal models to explan each CST parameter.

CST outcome parameter	Selected predictor parameters						
	status of eye (pre- or 3M sfter cataract surgery)	IOP-G	AL	Kerato	CCT	Age	Sex (male)
A1 Time [ms]		0.048		-0.003			
A1 Length [mm]				0.001	0.000		
A1 Velocity [m/s]		-0.002		0.000	0.000		
A2 Time [ms]		-0.028					
A2 Length [mm]					0.002		
A2 Velocity [m/s]	-0.023	0.005	-0.016		0.000		
HC Time [ms]	-0.151					-0.011	
Peak Dist. [mm]	0.080	-0.026	0.101	0.002		0.006	
Radius [mm]		0.063		0.008	0.004		
A1 Def. Amp. [mm]				0.000	0.000	0.000	-0.003
A2 Def. Amp. [mm]	-0.023	-0.005			0.000		-0.033
Def. Amp. Max [mm]	0.032	-0.013	0.022			0.003	

Number in each cell represents the coefficient value.

### Change in CST parameters pre- vs. 3m post-cataract surgery between the corneal incision and sclera-corneal incision groups

No significant differences were found for the changes in CST parameters between the corneal incision group and sclera-corneal incision group (all p>0.05, Welch’s *t*-test): see Table 2.

**Table 2:** Comparisons of the change in parameters between the corneal incision and sclera-corneal incision groups.

	Corneal incision group (N = 37) (%)	Sclero-corneal incision group (N = 22) (%)	P value (Welch's t-test)
IOP-G	-24.0±17.3	-7.9±28.3	0.002
IOP-CT90A	-13.1±13.2	-16.4±15.8	0.412
CST parameters			
IOP-CST	-5.3±17.5	-19.2±30.0	0.058
A1 Time	-1.5±3.9	-1.6±2.4	0.921
A1 Length	1.2±6.2	-0.6±4.5	0.175
A1 Velocity	4.2±11.4	7.2±8.2	0.236
A2 Time	0.9±4.1	0.3±4.0	0.584
A2 Length	2.2±13.0	1.0±19.7	0.806
A2 Velocity	11.3±22.7	10.5±22.6	0.894
HC Time	0.7±2.1	-1.1±3.5	0.644
Radius	-5.3±9.5	-1.8±8.8	0.159
Peak Distance	3.0±3.3	3.3±2.5	0.714
A1 Def. Amp.	0.1±7.7	0.1±3.5	0.982
A2 Def. Amp.	-0.6±13.5	0.4±18.2	0.803
Def. Amp. Max	6.7±6.8	6.2±6.0	0.746

Values in each cell represent mean ± SD (%).

## Discussion

In the current study, ocular biomechanics were evaluated before and after cataract surgery, using CST. IOP-G was observed to be significantly lower following cataract surgery. However, cataract surgery significantly affected a number of CST parameters (pre-surgery compared to 3m post-surgery), namely, A2 Velocity (coefficient = -0.023 for 3m-post operation), A2 deformation amplitude (-0.023), HC Time (-0.15), maximum deformation amplitude (0.032) and peak distance (0.080), adjusted for the change in IOP-G. Previously Ruão et al. showed an IOP-independent biomechanical change in A2 Velocity only after cataract surgery [24]. The authors showed that changes in IOP and CST parameters (post-surgical minus pre-surgical) were correlated. In the present study, we used sensitive statistical modeling to make an appropriate adjustment for IOP-G and other confounding variables. This may explain why we found that more biomechanical parameters (A2 Velocity, A2 deformation amplitude, HC Time, maximum deformation amplitude and peak distance) were significantly changed after cataract surgery.

According to the principles of non-contact tonometry, A1 Time strongly correlates with IOP [14, 25], but the change in A1 Time following surgery was not statistically significant. Furthermore, IOP-G and IOP-CT90A were significantly lower after cataract surgery, whereas IOP-CST was not. This is probably due to methodological differences between devices and the importance of each patient’s ocular biomechanics on the measurement principle. The CT-90A detects a flattening of the cornea by reflection of an installed infrared light while GAT measures IOP using the Imbert-Fick law. However, CST measures IOP based on a series of images that capture corneal deformation.

Corneal biomechanical change was demonstrated to be adjusted for change in IOP-G by linear modeling. This is a very interesting result and may suggest that any measured decrease in IOP (observed using GAT or CT-90A) might actually result from a change in corneal biomechanics. However, it is impossible to determine–from the current results–whether IOP-G decreased after cataract surgery due to a change in corneal biomechanics, a real change in IOP, or a combination of both. Nonetheless, it is also worth noting that IOP-CST was not significantly different following surgery, although it was lower in absolute terms. Similarly, Hassan et al. [22] reported that IOP readings obtained by CST were not significantly different after laser in situ keratomileusis and photorefractive keratectomy surgery, despite CCT decreasing.

Many CST parameters are correlated with IOP-G, as indicated in previous reports [14, 25]; therefore it is not surprising to see IOP-G selected as a predictor in a number of linear models. For example, the optimal model to describe A2 Velocity included IOP-GAT; A2 Velocity is correlated to IOP-G [14, 25] largely because corneal movement during its returning phase is faster when IOP-G is high [14, 25]. Nonetheless, eye status (coefficient = -0.023 for 3m-post operation) was also included in the model to explain A2 Velocity, which suggests A2 velocity is faster after cataract surgery despite a decrease in IOP-G. Interestingly, in eyes with trabeculectomy [28] as well as eyes with cataract surgery [24], A2 velocity quickens after surgery. The first applanation may reflect collagen fiber elasticity, whereas the second applanation may be related to corneal viscoelasticity [28]. Therefore, we suggest that eye surgery with direct invasion of the anterior chamber may cause viscoelasticity changes.

**Fig 3:**
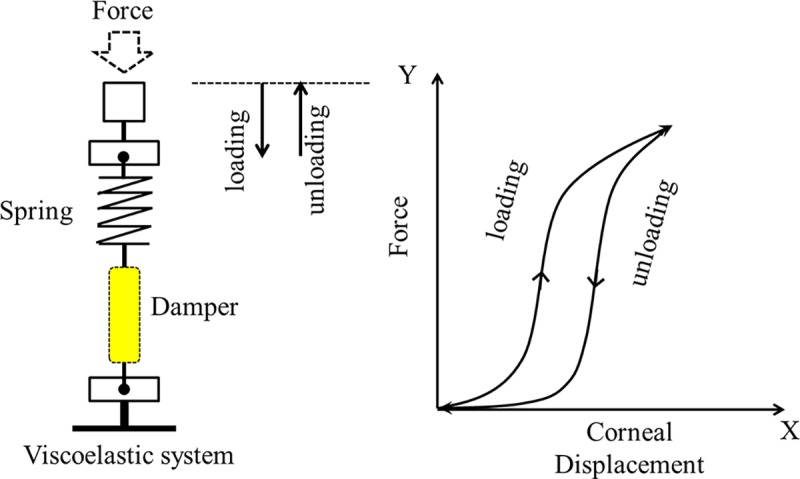
Depiction of the viscoelastic system containing an elastic spring and a viscous damper. The elastic hysteresis phenomenon is observed when cylcic loading is applied to a viscoelastic system. The loading and unloading deformation curves are called a hysteresis loop.

The viscoelastic property of the cornea can be described by a system that contains an elastic spring and a viscous damper (Fig 3) [29, 30].

**Fig 4:**
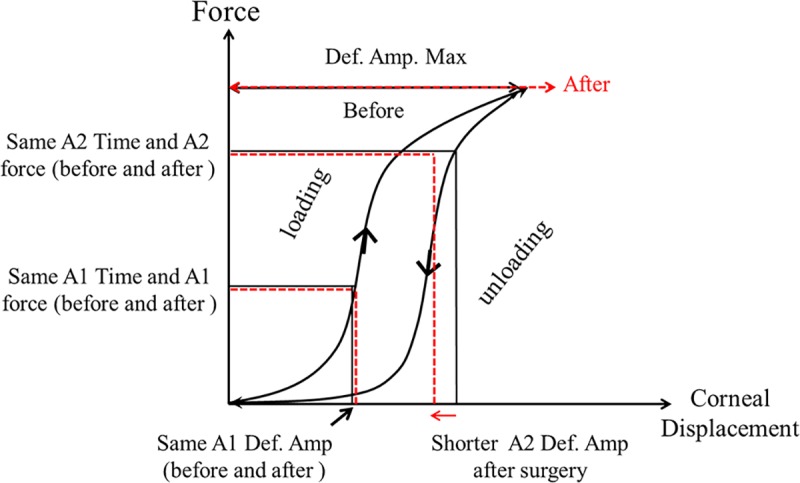
A change in the hysteresis loop may explain biomechanical changes to the eye after cataract surgery.

In this system, a difference occurs in the loading and unloading phases because part of the energy is dissipated by the system’s hysteresis. The relationship between the applied force and the magnitude of the displacement during loading and unloading periods are drawn as hysteresis curves (Fig 3). The area surrounded by these loading and unloading curves represents the hysteresis. The red arrows and lines in Fig 4 represent the impact of cataract surgery on these hysteresis curves. As maximum deformation amplitude increases, the apex of the loading and unloading curves moves in a rightwards direction. A1/A2 Time and A1 deformation amplitude do not change, but A2 deformation amplitude decreases. Thus, the corneal apex becomes longer due to an increased maximum deformation amplitude after cataract surgery, however, because A2 Time does not change whilst A2 deformation amplitude shrinks, A2 Velocity becomes faster following surgery.

The right shift of maximum deformation amplitude may result from a weakening of the corneal tip / spring in the viscoelastic system. Likewise, a weakened damper may result in a faster corneal movement from maximum deformation at HC Time to the A2 deformation amplitude point, because the energy applied to the eye (air puff) is poorly absorbed. Thus, an increase in A2 Velocity could be due to a weakened spring and/or damper in the corneal viscoelastic system after cataract surgery (Figs 3 and 4).

The black line shows the hysteresis loop before cataract surgery. The red dotted line shows the parameter changes after cataract surgery.

Reasons for this weakening after cataract surgery are unknown, however, elevation of IOP during cataract surgery may play a role. In a cataract operation, IOP in the anterior chamber is known to increase by roughly 50 to 60 mmHg while IOP in the vitreous cavity increases by approximately 40 mmHg [31]; this, in turn, could result in a stretching and distortion of ocular tissue.

A change in the biochemical environment, following cataract surgery, is another possible explanation for a weakened spring or damper phenomenon. Siriwardena et al., reported that the induction of anterior chamber flare was significantly higher in a cataract surgery group compared to a trabeculectomy group at 3 months post-surgery [32]. Furthermore, concentrations of monocyte chemoattractant protein-1 and growth factors are known to remain higher as long as 1–2 years after cataract surgery compared to before the operation [5, 33]. Finally, the wound healing process that occurs at the incision cite of cataract surgery may also have an influence on the biomechanical properties of the cornea. Interestingly, however, we observed no significant difference in CST parameters between the surgical and incision groups (corneal vs sclera-corneal incision group), as shown in Table 2. We observed a significant difference in IOP-G lowering between the corneal and the sclero-corneal incision group (-24.0% vs -7.9%). One previous study showed no significant difference between the two groups [34], while a different study suggested higher IOP reduction in the corneal incision group (-1.5 mmHg vs -0.6 mmHg) [35], which is consistent with our result.

Other studies have explored corneal biomechanical changes following cataract surgery. When ORA was used to measure such alterations, CH and CRF were reported to return to their pre-operative status within 3 months [7–9]. It is of great interest that some CST parameters were observed to remain significantly different, even at 3 months after surgery. The reason for this is not clear, but it is possible that CST can capture corneal biomechanical properties more sensitively than ORA is able to. A further investigation should be carried out shedding light on this finding; in particular, it would interesting to take both ORA and CST measurements at time periods longer than 3 months after surgery, such as 6 months and 1 year after surgery. Indeed this points to a limitation of the current study since CST was not conducted again after 3 months we are unable to judge whether the observed changes to corneal biomechanics are reversible or not. A second limitation of our study, of course, is that there is no method to measure ‘real’ IOP, noninvasively. Therefore we are unable to determine whether cataract surgery truly reduces IOP or whether measured IOP-G reductions are due to a change in corneal biomechanical properties.

## Conclusion

Corneal biomechanical properties, as measured with CST, were observed to change up to 3 months after cataract surgery, adjusted for a reduction in IOP-G.

This confirms results given in a previous report [24] and adds new evidence that a change in the hysteresis loop may explain biomechanical changes to the eye after cataract surgery.

## Supporting Information

S1 Table All dataset. The dataset contains data for all 59 patients included in the study.
(XLSX)
